# Brewing a Craft Belgian-Style Pale Ale Using *Pichia kudriavzevii* 4A as a Starter Culture

**DOI:** 10.3390/microorganisms11040977

**Published:** 2023-04-09

**Authors:** Vogar Leonel Nieto-Sarabia, Guiomar Melgar-Lalanne, Christian Bryan Ballinas-Cesatti, Fernando Abiram García-García, Jorge Alberto Jose-Salazar, César Mateo Flores-Ortiz, Eliseo Cristiani-Urbina, Liliana Morales-Barrera

**Affiliations:** 1 Departamento de Ingeniería Bioquímica, Escuela Nacional de Ciencias Biológicas, Instituto Politécnico Nacional, Av. Wilfrido Massieu s/n, Unidad Profesional Adolfo López Mateos, Ciudad de México 07738, Estado de México, Mexico; 2 Instituto de Ciencias Básicas, Universidad Veracruzana, Av. Castelazo Anaya s/n, Industrial Ánimas, Xalapa 91190, Veracruz, Mexico; 3 Unidad de Biotecnología y Prototipos, Facultad de Estudios Superiores-Iztacala, Universidad Nacional Autónoma de México, Los Reyes Iztacala, Tlalnepantla 54090, Estado de México, Mexico; 4 Laboratorio Nacional en Salud, Facultad de Estudios Superiores-Iztacala, Universidad Nacional Autónoma de México, Los Reyes Iztacala, Tlalnepantla 54090, Estado de México, Mexico

**Keywords:** beer production, beer guidelines, alcoholic fermentation, non-*Saccharomyces* yeasts, *Pichia kudriavzevii*, mycotoxins

## Abstract

There is an expanding market for beer of different flavors. This study aimed to prepare a craft Belgian-style pale ale with a non-*Saccharomyces* yeast. *Pichia kudriavzevii* 4A was used as a sole starter culture, and malted barley as the only substrate. The ingredients and brewing process were carefully monitored to ensure the quality and innocuousness of the beverage. During fermentation, the yeast consumed 89.7% of total sugars and produced 13.8% *v*/*v* of ethanol. The product was fermented and then aged for 8 days, adjusted to 5% *v*/*v* alcohol, and analyzed. There were no traces of mycotoxins, lead, arsenic, methanol, or microbiological contamination that would compromise consumer health. According to the physicochemical analysis, the final ethanol concentration (5.2% *v*/*v*) and other characteristics complied with national and international guidelines. The ethyl acetate and isoamyl alcohol present are known to confer sweet and fruity flavors. The sensory test defined the beverage as refreshing and as having an apple and pear flavor, a banana aroma, and a good level of bitterness. The judges preferred it over a commercial reference sample of Belgian-style pale ale made from *S. cerevisiae*. Hence, *P. kudriavzevii* 4A has the potential for use in the beer industry.

## 1. Introduction

During a great part of human history, fermentation has served as a means of preserving food by modifying its nutritional properties. Another use of fermentation processes today is to generate beverages with sensory characteristics that are pleasant for consumers [1]. One particularly popular product of alcoholic fermentation is beer, which is traditionally elaborated with *Saccharomyces cerevisiae* yeast and barley.

Although the precise origin of alcoholic fermentation is not certain, the most conclusive archeological evidence dates back to the period from 5500 to 3100 BC in Egypt. Since then, beer and wine have played an important role in the development of socio-economic and political relations between civilizations [2]. During much of human history, beer-making was considered an art mainly revolving around spontaneous fermentation. In the last century, science has helped the beer industry to evolve by establishing fermentation technology with controlled and reproducible processes capable of guaranteeing the quality of the beverage [3].

The main raw materials that contribute to the aroma, flavor, and appearance of beer are water, malt, hops, and yeast. Water is the principal component of the final product. Its properties, including pH, influence the enzymatic reactions throughout the brewing process [4]. The second most abundant element is malt, which is usually derived from barley but can also be obtained from wheat, oats, and rye [5], as well as less conventional cereals (e.g., sorghum, rice, and corn) and pseudo-cereals (e.g., amaranth and quinoa) [6]. The malting of grains allows their enzymes to convert starches into various types of fermentable sugar that serve as the substrate of yeast [5]. Malted grains are stored and must be carefully inspected to identify fungi capable of producing mycotoxins [7]. Hops, on the other hand, are bacteriostatic agents that contain resins, which provide the typical bitter flavor of beer [4]. In the brewing process, yeast is the active ingredient responsible for fermentation and thus the generation of ethanol, CO_2_, and other compounds involved in creating pleasant sensory properties and good-quality beer [8,9].

*S. cerevisiae* and *S. pastorianus* (syn. *S. carlsbergensis*) are yeast species commonly used for the industrial production of beer [9]. The majority of non-*Saccharomyces* species utilized in the elaboration of this beverage have been isolated from spontaneous fermentation. They include species from the genera *Brettanomyces* [10], *Pichia kluyveri*, *P. anomala*, *P. kudriavzevii*, *Candida stellata*, *Torulaspora delbrueckii* [11], *Candida krusei*, *Cryptococcus kuetzingii*, and *Rhodotorula mucilaginosa* [12]. However, few of them have been employed in the industrial preparation of beer, either alone or in combination with a *Saccharomyces* yeast. Since these non-*Saccharomyces* yeasts can impart pleasant spicy, flowery, and fruity aromas and flavors [9,10,11], they represent an opportunity for innovation in the growing beer industry.

Beer is the third most popular drink in the world after water and tea, with approximately 1.91 billion hectoliters produced in 2019 [13]. The global market was estimated to have a value of US$623.2 billion in 2020 [14], considering the total amount of industrial and craft beer brewed.

The elaboration and consumption of craft beer have increased globally during the last few decades [15]. In Mexico, for example, there was a growth of 8% from 2019 to 2020 [16]. Craft beer is popular because of its particular sensory properties, which are commonly derived from the addition of spices, herbs, flowers, and fruit [15]. Starter cultures with unconventional yeasts have also been used to attain innovative flavors appealing to the sensory experience of consumers [17].

Craft beer is characterized by small-scale production in the absence of pasteurization and filtering. Compared to industrially produced beer, it is richer in nutritive compounds, which gives it a more limited shelf life [7]. The process of preparation of craft beer typically lacks strict quality control and reproducibility of the sensory profile [15]. It should be a priority to maintain a high quality of raw materials in the malting and brewing processes of craft beer and to monitor the presence of pathogenic microorganisms, heavy metals, and toxins that can adversely affect the beverage and, consequently, the health of consumers [4,7].

The aim of the current contribution was to elaborate a craft Belgian-style pale ale based on a strictly reproducible brewing process using *Pichia kudriavzevii* 4A yeast as the starter culture and malted barley as the only substrate. This non-*Saccharomyces* yeast was employed to achieve a novel flavor attractive to consumers in the growing beer market. The study successfully achieved a beverage innocuous to consumer health with a novel flavor that was more attractive to the panel of judges than the reference sample of a commercial Belgian-style pale ale.

*Pichia kudriavzevii* 4A, presently isolated from the soil of a sugar cane field, exhibits an elevated capacity for the utilization of fermentable sugars from malted barley to generate ethanol [18,19]. In spite of being previously used as a starter monoculture for the preparation of traditional rice beer [20], it has not, to our knowledge, constituted a starter monoculture for the production of beer based on malted barley as the only source of carbon.

The ingredients and the brewing process were carefully monitored to ensure the quality and innocuousness of the resulting craft beer, which according to the physicochemical and microbiological analysis, complied with national and international guidelines in relation to adequate physical characteristics and chemical content. Initially, a physicochemical analysis was conducted on the barley, followed by an examination of the changes that took place in the wort during fermentation. Subsequently, the physical, chemical, and microbiological characteristics of the final product were determined, and the content of mycotoxins was evaluated. Finally, a sensorial test was carried out to explore the specific flavors of the beer and the overall preference of the judges.

## 2. Materials and Methods

### 2.1. Barley and Germination

The six-row barley (*Hordeum vulgare* L.) that served as the substrate was obtained from the municipality of Metztitlán (Hidalgo, Mexico) 20°35′12.8″ N, 98°45′31.8″ W. To verify the quality of the barley, we submitted it for a physicochemical evaluation, including tests for the presence of toxins. Once its suitability was established, the study proceeded to the malting phase. For germination, 200 g of barley seeds were cleaned with distilled water before being left to soak in water for 24 h. Upon completion of this time, the seeds were placed on trays and kept in a room with 90% relative humidity at 20 °C for 120 h [21,22]. Samples were taken of the germinated seeds at 0, 3, 4, and 5 days to determine the level of glucose and select the best germination time.

### 2.2. Malting

The seeds were germinated for 4 days and then dried in an oven at 60 °C for 72 h. Subsequently, they were ground in a hammer mill (Glen Creston, London, UK) and sifted to obtain a particle size under 0.5 mm. One part of the malt was maintained in plastic-coated Kraft paper bags at room temperature for the posterior preparation of the beer wort, while the other part was subjected to an analysis of humidity and the possible presence of toxins.

### 2.3. Elaboration of the Sweet Beer Wort

A suspension of the germinated barley malt in water was left for 4 days (1:15 *w*/*v*), then heated to 65 °C under constant stirring at 140 rpm for 1 h. After being separated by decantation, the particles of the suspension were filtered with the Whatman paper (grade 42). The glucose concentration in the filtrate (sweet wort) was quantified to allow for the formulation of the final culture medium (supplemented wort).

### 2.4. Formulation of the Supplemented Wort

A sufficient amount of the sweet wort was added to the culture medium to ensure an initial glucose concentration of about 12 g L^−1^. The suspension was supplemented with salts (3.0 g L^−1^ (NH_4_)_2_SO_4_, 1.0 g L^−1^ KH_2_PO_4_, 0.3 g L^−1^ MgSO_4_·7H_2_O, 0.01 g L^−1^ KCl, 0.05 g L^−1^ CaCl_2_, and 0.0001 g L^−1^ FeCl_2_·6H_2_O) and 0.03 g L^−1^ of yeast extract, then sterilized in an autoclave [23]. The glucose concentration and total sugars (measured as maltose) were determined in the supplemented wort to establish the initial value in the substrate and the final value after fermentation. The supplemented wort was used for the propagation of the culture inoculum as well as the brewing of the beer.

### 2.5. Isolation of the Yeast and Preparation of the Inoculum

**Figure 1:**
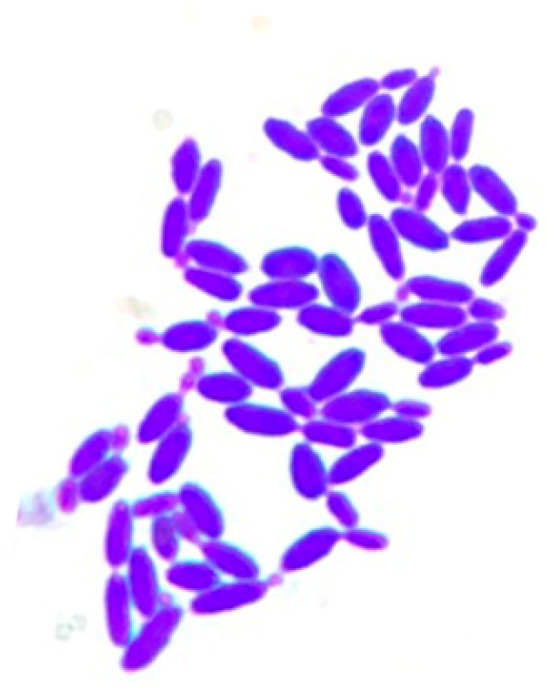
Optical micrograph of *Pichia kudriavzevii* 4A (40×).

*Pichia kudriavzevii* 4A (Figure 1) was isolated from the soil of a sugar cane field in the municipality of Úrsulo Galván, Veracruz, Mexico.

It was identified by molecular tests [19] and conserved in a YPG medium consisting of 10 g L^−1^ yeast extract, 20 g L^−1^ dextrose, 20 g L^−1^ casein peptone, 20 g L^−1^ bacteriological agar, and distilled water [18,23]. For the preparation of the inoculum utilized in brewing, an inoculation loop of yeast conserved in solid YPG medium was put in each of various 500 mL Erlenmeyer beakers containing 120 mL of medium plus the supplemented wort. The beakers were placed on an orbital shaker operating at 140 rpm for 72 h. Subsequently, the suspension was centrifuged at 3500 rpm for 15 min to recover the cells. To each of the various 1 L Erlenmeyer beakers with 250 mL of culture medium (based on the supplemented wort), a suitable quantity of the cellular pellet was added in order to attain an initial biomass concentration of 0.1 g L^−1^, followed by incubation at 140 rpm and room temperature for 72 h. The content of these beakers was centrifuged under the previously described conditions, and the biomass was recovered to be inoculated into the fermenter.

### 2.6. Elaboration of the Beer

A 30 L stainless-steel fermenter was washed with a solution of 0.5 M NaOH at 80 °C for 15 min and rinsed with an abundance of water. Another wash was carried out 24 h later with an acid-based food-grade sanitizer (Star San, Five Star, Arvada, CO, USA), leaving the apparatus ready to receive the culture medium.

After mixing 23 L of the enriched wort (without sterilization) with 150 g of hops (SAAZ, CZE) and boiling the liquid for 1.5 h, it was passed through a plate heat exchanger at 91 °C for 30 min to pasteurize the medium. When coming out of the heat exchanger at 25 °C, it was transferred directly to the fermenter. Under aseptic conditions, approximately 23 g of the biomass was then inoculated into the solution to form an initial biomass concentration of around 1 g L^−1^. Air was immediately injected into the mixture by means of a stone diffuser (5 mm in diameter) until saturating it with oxygen (about 7 mg O_2_ L^−1^).

Upon completion of 7 days of fermentation at 25 °C, the biomass was extracted by sedimentation, quantified, and discarded. A sample of the liquid was evaluated for ethanol production and substrate consumption. The ethanol concentration was determined in the rest of the liquid, which was divided into two fermenters, and the degree of alcohol was adjusted to approximately 5% *v*/*v* by adding water. The liquid in the fermenters was left to mature at 5 °C for 1 day followed by 15 °C for 6 days. Then the liquid was transferred to a stainless-steel barrel, and the concentration of ethanol was adjusted again to 5% *v*/*v*. The medium was injected with CO_2_ to maintain the barrel at a pressure of 15 psia for 24 h before being left to stand for another 24 h.

Finally, the beverage was bottled under aseptic conditions in amber glass bottles previously washed with sanitizer (Star San, Five Star, Arvada, CO, USA). The resulting Belgian-style pale ale was subjected to microbiological and physicochemical analysis, including an examination of possible toxins. Once the beverage was found to be innocuous, it was submitted to sensory tests.

### 2.7. Physicochemical Analysis of the Barley

The raw barley (before germination) was assessed physically for aroma, aspect, impurities, and weight per hectoliter and for viability by the tetrazolium method [24,25]. It was subjected to proximate analysis to establish the percentage of humidity, raw protein, lipids, ash, and raw fiber in accordance with the methods of the AOAC (934.01, 2001.11, 920.39, 942.05, and 962.09, respectively) [26]. The quantity of the nitrogen-free extract was calculated as the remaining amount, subtracting the aforementioned values of the chemical analysis from 100%.

### 2.8. Evaluation of the Wort: Glucose, Total Sugars, Biomass, and Ethanol

Glucose was quantified in the wort by the enzymatic method of the glucose oxidase/peroxidase test [27], and the total sugars by the sulfuric acid method, modified by DuBois [28]. The biomass was determined by dry weight and the ethanol concentration with an alcohol hydrometer.

### 2.9. Inspection of the Beer for Quality

The total coliform content was established by the most probable number (MPN) method [29] to verify the quality of the final product and its compliance with regulations. The pH of the beer was assessed with a potentiometer, and its acidity by the volumetric method was quantified as lactic acid [30]. The ethanol concentration was determined as a percentage of volume at 20 °C by using an alcohol hydrometer [31]. With the prescribed methods, the content of methanol, ester, aldehyde, and higher alcohols was analyzed by gas chromatography on a Perkin Elmer apparatus (Clarus 480, Perkin Elmer, Whatman, MA, USA) [32], and lead and arsenic were quantified on a Perkin Elmer atomic absorption spectrophotometer (PinAAcle 900H, Perkin Elmer, Whatman, MA, USA) with a flow injection system (FIAS 100, Perkin Elmer, Whatman, MA, USA) [33]. All the samples were read on the same atomic absorption spectrophotometer in the flame mode to assess the level of lead and by the hydride generation technique to examine the level of arsenic.

### 2.10. Analysis of Toxins

The possible presence of twelve mycotoxins in samples of barley, malted barley, and the final product was evaluated by the Biogeochemical Lab and the National Lab of Health in the Faculty of Graduate Studies Iztacala of the Universidad Nacional Autónoma de México. For the barley and malted barley, the process of extraction of the toxins began by grinding each sample. In total, 10 g of powder of each sample was added 30 mL of acetonitrile: ethyl acetate: formic acid in a proportion of 10:8:0.18 (*v*/*v*/*v*), and then the mixture was shaken at 150 rpm for 30 min in a mechanical orbital agitator. The solution was passed through F2041 filter paper, and the residue was evaporated to dryness under reduced pressure. Regarding the final product, the extraction was performed by the methodology described by Pascari et al. [34]. Each extraction obtained from the barley, malted barley, and beer was resuspended in 1.5 mL of methanol and stored at 4 °C for posterior analysis. The following toxins were identified in reverse phase in accordance with the proposed method [35] using HPLC (1260 Infinity-ESI-TOF-MS (6230) Agilent Technologies): T-2, HT-2, diacetoxyscirpenol (DAS), neosolaniol (NEO), fumonisin B_1_ (FB_1_), zearalenone (ZEN), and aflatoxins (AFB_1_, AFG_1_, AFB_2_, and AFG_2_). A Zorbax Eclipse Plus C18 column (Agilent, Santa Clara, CA, USA) (1.8 μm, 2.1 × 100 mm, with an injection volume of 10 µL) was utilized for the separation of the compounds. Ochratoxin A (OTA) and deoxynivalenol (DON) were identified and quantified by HPLC (Agilent 1100 series-FLD LS 50B Perkin Elmer) and HPLC-DAD (Agilent 1100 series) in reverse phase, following the procedures described by Wilson [36] and Thammawong et al. [37], respectively, with a Discovery C-18 column and an injection volume of 20 µL.

### 2.11. Sensory Analysis of the Beer

Once the innocuousness of the consumption of the pale ale brewed with *P. kudriavzevii* 4A was assured, it was submitted to sensory analysis. According to the Eurachem Guide on Selection, Use and Interpretation of Proficiency Testing (PT) Schemes by Laboratories [38], reference samples serve as control samples in sensorial tests. A commercial Belgian-style pale ale was herein used as the reference sample.

A comparative sensorial study between a new beer and a commercial beer as a reference sample has the aim of determining the preference of the consumer. By employing this methodology, one study developed a new beer with a wort of fruits that conferred a novel flavor [39], and another study elaborated a new beer with a non-*Saccharomyces* yeast strain, which was *Cyberlindnera subsufficiens* C6.1 [40]. Still, another study incorporated new ingredients into the process of producing a beer to diminish the fermentation time, then conducted a comparative sensorial test [41].

For such sensorial studies, it is common to use a panel of judges to determine the flavor and overall preference, whether they be beer consumers [42,43] or expert beer tasters [44]. The present study based the sensorial test on 50 untrained judges. Two types of tests were a descriptive profile and a sensory assessment. Regarding the former, the panelists evaluated (on a scale of 0–8) seven characteristics considered important for the acceptability of the beverage (turbidity, refreshing taste, brightness, fruitiness, spiciness, bitterness, and flavor). The results are reported with a radar chart. Concerning the sensory assessment, the panelists were asked to express their overall preference for one of two Belgian-style pale ales: the product of the present study and the reference sample.

### 2.12. Statistical Analysis

The tests to assure the quality of the barley, barley malt, and beer were performed at least in triplicate (*n* ≥ 3). The sensory analysis was based on the opinion of 50 panelists (*n* = 50). The statistical analysis was conducted with GraphPad Prism^®^ version 9.1 (GraphPad Software, San Diego, CA, USA), considering significance at *p* < 0.05.

## 3. Results and Discussion

### 3.1. Evaluation of the Physicochemical Characteristics of the Barley

The results of the physical and chemical composition analysis of the barley seeds are shown in Table 1.

The evaluation of aroma and color in the dry barley grain revealed the adequacy of this raw material, as practically all grains were found to be whole, fresh, and without any notable microbial growth. The impurities detected were a few herbs, broken seeds, and small stones, but no insects. The weight per hectoliter was 57.17 kg hL^−1^, which complies with the guidelines. The greater the weight per hectoliter, the higher the yield and quality of the barley [24]. The percentage of viability (87.33%) was above the established minimum limit [25], indicating good handling during the harvest and adequate storage of the grain. The proper germination of the seeds leads to a better yield of the malt [46].

The chemical composition analysis of the dry matter of the barley revealed values close to those reported in the literature. The ash content of the barley grain was low (approximately 2.82%) and mostly composed of inorganic compounds related to the mineral content of the soil and the fertilizers used during cultivation [45].

In the barley, there was a small amount of lipid (3.11 ± 0.08%), which is known to affect the flavor and stability of the beer foam [3]. If bound to proteins, lipids stabilize and improve the foam of the final product. If not, they diminish the quality of the foam [45]. Crude fiber, mainly cellulose, is normally about 6% of the weight of the grain. It was herein found to be 6.29 ± 0.01%. Regarding protein, the 11.72 ± 0.03% encountered fell within the 8–13.5% range of the guidelines. Among the principal proteins in barley grain are albumins, globulins, prolamins, and glutenin. Whereas albumin and globulins are important for the colloidal characteristics of beer, and the formation of foam, prolamins, and glutenin are plant storage proteins [4].

Carbohydrates comprised 76.10 ± 0.2% of the barley seeds. Since starch makes up 72.8 to 82.8% of total carbohydrates (and up to 63% of the seed), a greater quantity of carbohydrates implies a greater content of starch, which is crucial for the malting process. Starch is hydrolyzed into maltose and other simple sugars that constitute the substrate of the yeast. Among the other main carbohydrates of barley are hemicellulose and gum, which provide viscosity to the beer wort [4,45,47].

The humidity of the raw seeds was 8.22%. A grain conserved in adequate conditions should have less than 12% humidity. While the present level of humidity was sufficient to favor the activity of certain desirable enzymes in the grain, it prevented the growth of fungi that can deteriorate barley and produce toxins harmful to consumers [34]. Given its importance, the humidity was also analyzed in the malted barley. The 4.48% found is below the maximum recommended value (~5%) [48]. After comparing the current results with the guidelines and reported values (Table 1), the barley was deemed suitable as raw material for the production of malt.

### 3.2. Evaluation of Glucose in the Grain

Yeasts are capable of fermenting simple sugars, including glucose and fructose. Thus, for some yeasts (e.g., *S. cerevisiae*) to be able to carry out fermentation and generate ethanol, it is essential for complex substrates rich in starch (e.g., barley seeds) to be previously hydrolyzed into simple sugars [49]. The malting of barley, a process that takes 3–7 days [48], allows the enzymes of the grain to hydrolyze starch, proteins, and nucleic acids in the smallest molecules into a form easily utilized by the yeast for the elaboration of beer [47].

In the current study, the change in the glucose concentration of the barley seeds was monitored during germination at distinct times (0, 3, 4, and 5 days) to define the germination time (Table 2) suitable for the adequate growth of *P. kudriavzevii* 4A during the preparation of the supplemented wort.

The quantification of glucose revealed that its concentration increased over 13-fold in the first 3 days of germination, resulting from the hydrolysis of the starch content of the barley seeds. The two-way ANOVA analysis by the Tukey method indicated a significantly greater (*p* < 0.05) glucose concentration of the grain between each day from day 0 to days 3 and 4 of germination. On the other hand, the increment in the glucose concentration during days 4 and 5 did not constitute a significant difference from the value of the previous day. Consequently, the malt was harvested on day 4 for its posterior use to produce the supplemented wort.

### 3.3. Evaluation of Biomass, Sugars, and Alcohol Content before and after 7 Days of Fermentation

Among the factors that affect fermentation are the presence of mineral salts, medium acidity, sugar type and concentration, dissolved oxygen level, pH, incubation time, and temperature, among others. Additionally, the nature of the substrate and the microorganism have a significant influence on the outcome of the fermentation [50]. In this study, it was observed that the enriched wort contained an initial concentration of total sugars of 125 g L^−1^ (measured as maltose), of which glucose comprised 9.6% (12 g L^−1^). At the end of the 7-day fermentation period, the necessary samples were taken from the fermenter to quantify the growth of the yeast, the consumption of the substrate (glucose and total sugars), and the production of ethanol. *P. kudriavzevii* 4A consumed 112.10 ± 1.41 g L^−1^ of total sugars, which represents an 89.7% efficiency in the consumption of the substrate. The yeast completely consumed glucose, generating 109.18 ± 1.3 g L^−1^ (13.84 ± 0.16 % *v*/*v*) of ethanol. The biomass produced by the yeast was 44.91 ± 0.60 g L^−1^ (Table 3).

From these results, it is possible to appreciate the extreme efficiency in the consumption of sugars and generation of ethanol by *P. kudriavzevii* 4A. This yeast has a proven capacity to employ various sources of carbon as substrate [20]. Additionally, *P. kudriavzevii* is reported to be osmotolerant and thermotolerant (capable of growing at up to 200 g L^−1^ of glucose and up to 40 °C) to produce and resist an elevated ethanol concentration (approximately 13% *v*/*v*), to be able to grow at a very low pH (less than 2), to resist the presence of toxic compounds such as 5-hidroxymethylfurfural (up to 1 g L^−1^), and to have activity as a killer yeast [19,51,52]. Compared to other microorganisms, the characteristics of *P. kudriavzevii* offer advantages in industrial processes. One important advantage is a decreased risk of microbiological contamination.

Once the first stage of fermentation ended, the yeast had become accustomed to an ethanol concentration of about 5% *v*/*v*. The wort was left to mature before continuing with the elaboration of the beer. The final ethanol concentration was 5.2% *v*/*v*.

### 3.4. Evaluation of Physicochemical and Microbiological Parameters of the Beer

The pleasure that a consumer obtains from beer is based on personal sensory perception. To evaluate this perception and assure the safety of the beverage, it is necessary to establish some parameters in relation to its composition and quality. The present Belgian-style pale ale was characterized and compared to the Mexican guidelines (Table 4).

The pH of beer (~4.5) [4] plays a crucial role in the colloidal stability of the product and its foam, as well as in the aroma, flavor, and sensation of the freshness of the beverage [20,55]. Additionally, its low pH serves to diminish the load of pathogens, coliform bacteria, and sporulated microorganisms [20]. A value under 4 may indicate the presence of bacteria and, at the same time, bring about a more bitter and metallic taste. At a pH above 4.5, beer can take on a soapy taste [55]. Another criterion for evaluating beer is total acidity as a measure of the content of organic acids, which confer sensory properties [56].

The allowable ethanol concentration of beer is 2–20% *v*/*v* [53]. A lower ethanol concentration characterizes light beer (<1% *v*/*v*) or non-alcoholic beer (0.0%) [15]. The standard for beer is 4–6% *v*/*v* [4].

The current results of pH (4.23) and total acidity (2.44 mg 100 mL^−1^) comply with the established guidelines and the intervals described in other studies [30,56]. The level of ethanol (5.2% *v*/*v*, equivalent to 41.03 g L^−1^ of ethyl alcohol) classified this pale ale in the intermediate range [31].

For comparative purposes, there are no known reports on the preparation of beer by using only barley as the substrate and *P. kudriavzevii* as the starter culture. The data on the resulting product are close to those published by Ghosh et al. [20], who elaborated a traditional rice beer with *Pichia kudriavzevii*, obtaining two products with an average total acidity of 2.3 mg 100 mL^−1^, a pH of 5.5, and an ethanol content of 6.4% *v*/*v*.

The compounds responsible for giving the predominant flavor and aroma to beer are higher alcohols, esters, aldehydes, and organic acids [17]. While a level of higher alcohols above 300 mg L^−1^ confers a strong and pungent flavor that could possibly be unpleasant [8], a concentration below this level can afford an attractive sweet flavor. By reacting with alcohol having different forms of acetyl Co-A, higher alcohols may also serve as precursors of esters, which generally furnish a pleasant fruity flavor [5]. According to previous reports, under certain conditions, *Pichia kudriavzevii* is capable of producing isoamyl alcohol (providing an alcoholic, vinous, or sweet aroma), ethyl acetate (affording fruity or solvent-like flavors), and a low concentration of isoamyl acetate (emitting a banana aroma) [11,57].

The oxidation of alcohol generates aldehydes during the storage of beer. Due to being intermediaries in the fermentation process (e.g., acetaldehyde) as well as products of thermal treatment during pasteurization (e.g., Strecker aldehydes), aldehydes are found in beer at the end of the fermentation process [58]. During the maturation of beer, some aldehydes are eliminated by means of carbonation (injection of CO_2_) [4]. Although not all impart notable flavors, certain aldehydes are related to a rancid flavor in the final product. Their presence always indicates the oxidation and aging of beer [58]. In the pale ale elaborated with *Pichia kudriavzevii* 4A, aldehydes were not detected (Table 4), which is typical of recently prepared beer with an adequate process of carbonation.

**Figure 2:**
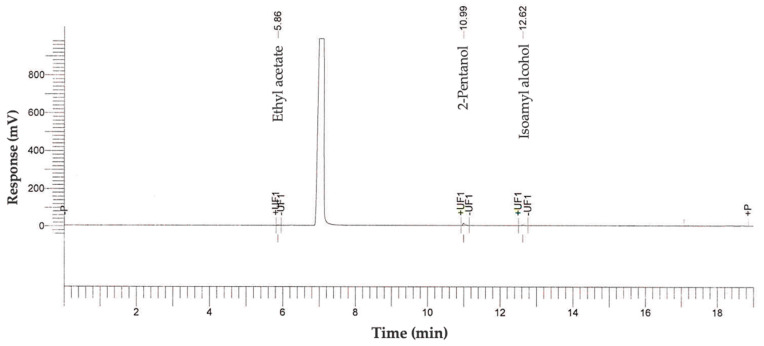
Chromatographic profile of methanol, esters, aldehydes, and higher alcohols of the beer prepared from *Pichia kudriavzevii* 4A.

The chromatograph that was recorded to quantify methanol, esters, aldehydes, and higher alcohols in the fermented beer is illustrated in Figure 2.

Of the total higher alcohols (140.95 mg 100 mL^−1^ anhydrous ethanol), isoamyl alcohol represented 77.5% and 2-pentanol 22.5%. These are known to increase the sensation of alcohol in the beverage as well as the aroma of fruit [8]. The ester detected in the final product is ethyl acetate (71.72 mg 100 mL^−1^ anhydrous ethanol). There is a relation between the production of isoamyl acetate and ethyl acetate. At a certain level, they produce a sweet and fruity aroma [57].

The Mexican guidelines do not consider limits on methanol, esters, aldehydes, and higher alcohols in a fermented (versus distilled) beverage. In the case of distilled alcoholic drinks, the limits established by the regulations are far above the present values. For higher alcohols, the maximum level is 500 mg 100 mL^−1^ anhydrous ethanol; for esters, 200 mg 100 mL^−1^ anhydrous ethanol; and for aldehydes, 40 mg 100 mL^−1^ anhydrous ethanol.

Although unconventional yeasts are used to diversify the array of aromas and flavors of fermented beverages such as beer, they can generate secondary products (e.g., methanol) harmful to consumer health [59]. Hence, special attention is required in examining fermentation products to avoid toxins.

The cell wall of the endosperm of barley contains up to 5% pectin, implying the existence of this compound in the raw grain and its malted form [60]. Certain pectolytic enzymes induce an increase in the methanol concentration during fermentation. They have been identified in some beers based on a thorough analysis of the entire brewing process [61]. In this study, methanol was not detected in the final product (Table 4).

Since any of the raw materials may contain heavy metals, an evaluation was performed to detect such metals and thus ensure quality control of the beer. Although heavy metals are generally found in low concentrations when derived from raw materials [7], higher concentrations may result from the filters used in the industrialized fermentation of alcoholic beverages. This is especially true with filters made of diatomaceous earth, which tend to release lead (Pb) and arsenic (As) and therefore compromise the safety of the product for consumers [62]. The Mexican norm sets 0.5 mg L^−1^ as the limit for Pb and 0.5 mg L^−1^ for As. The concentrations of Pb (<0.3 mg L^−1^) and As (<0.002 mg L^−1^) in the present beer were below those stipulated in the regulations. A positive factor in the elaboration of craft beer is the lack of filtration.

Finally, an evaluation was made of the possible microbiological contamination from the raw materials, equipment, and utensils employed in the brewing process. It is also common for contaminants to enter beer during bottling [7]. The total coliform bacteria herein detected was <1.1 (MPN/100 mL), which is within the permissible limits [54].

### 3.5. Analysis of Mycotoxins in the Beer

Given that the physicochemical and microbiological characteristics of the pale ale were within the established guidelines, an analysis of mycotoxins was carried out. The latter are low molecular weight compounds generated as secondary metabolites by some genera of filamentous fungi, including *Aspergillus*, *Penicillium*, and *Fusarium*. They appear in beer due to the inappropriate storage of raw materials (barley, malted barley, hops, and other ingredients) [34]. Mycotoxins can cause great economic losses and be harmful to public health. Among the harmful effects are hepatoxicity, carcinogenicity, nephrotoxicity, teratogenicity, infertility, and adverse gastrointestinal and immunosuppressive effects [63]. Moreover, mycotoxins are capable of negatively affecting the metabolic activity of the yeast, thus altering fermentation [7].

The presence of mycotoxins was analyzed in the barley, malted barley, and beer in order to identify any possible source of contamination and consequently assure the innocuousness of the final product for public consumption. Since each country has its own regulations in regard to the limits of mycotoxins permitted in a beverage, and these tend to vary greatly, the standard used to compare the results of the current contribution (Table 5) was that of the Food and Agriculture Organization of the United Nations [64].

The concentration of mycotoxins turned out to be below the detectable level, which indicates the obtention of an innocuous product and compliance with the international norm. Unfortunately, this type of study is rarely carried out on craft beers, representing a risk in the consumption of such beverages [65].

### 3.6. Sensory Evaluation

Though subjective, the sensory test establishes an important profile of the flavor of a food or beverage elaborated from raw materials. Apart from indicating the quality of a product and thus its possible acceptance by consumers, this assessment can discover its deterioration due to the time of storage and/or microbiological activity [66,67]. In order to obtain desirable attributes in a craft beer, it is necessary to maintain a balance between an attractive flavor and aroma. Among the factors capable of having a positive and negative effect are aldehydes, esters, lipids, organic acids, and higher alcohols [68,69].

**Figure 3:**
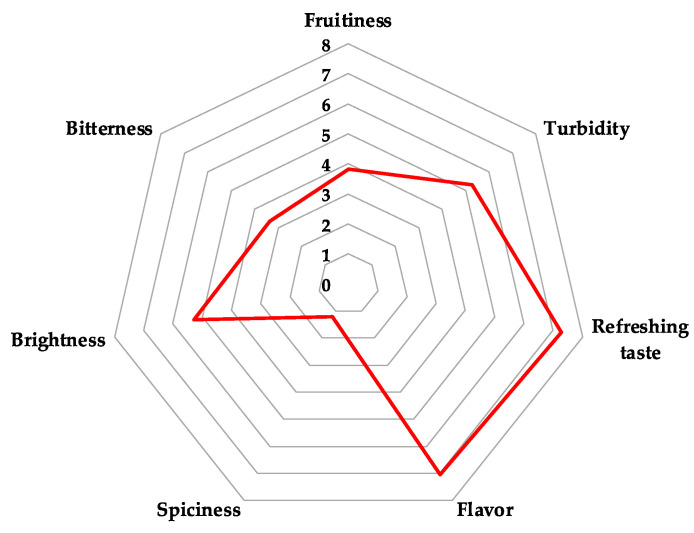
Evaluation of some attributes of the beer made with *P. kudriavzevii* 4A.

In the sensory evaluation of multiple attributes of the pale ale prepared with *P. kudriavzevii* 4A, the panelists qualified the beverage mainly as refreshing and flavorful, with an adequate level of bitterness and brightness (Figure 3). Regarding the spicy notes of the beverage, a fruity taste was predominantly perceived. Turbidity was an evident quality typical of craft beers because they are not filtered to eliminate all the biomass.

**Figure 4:**
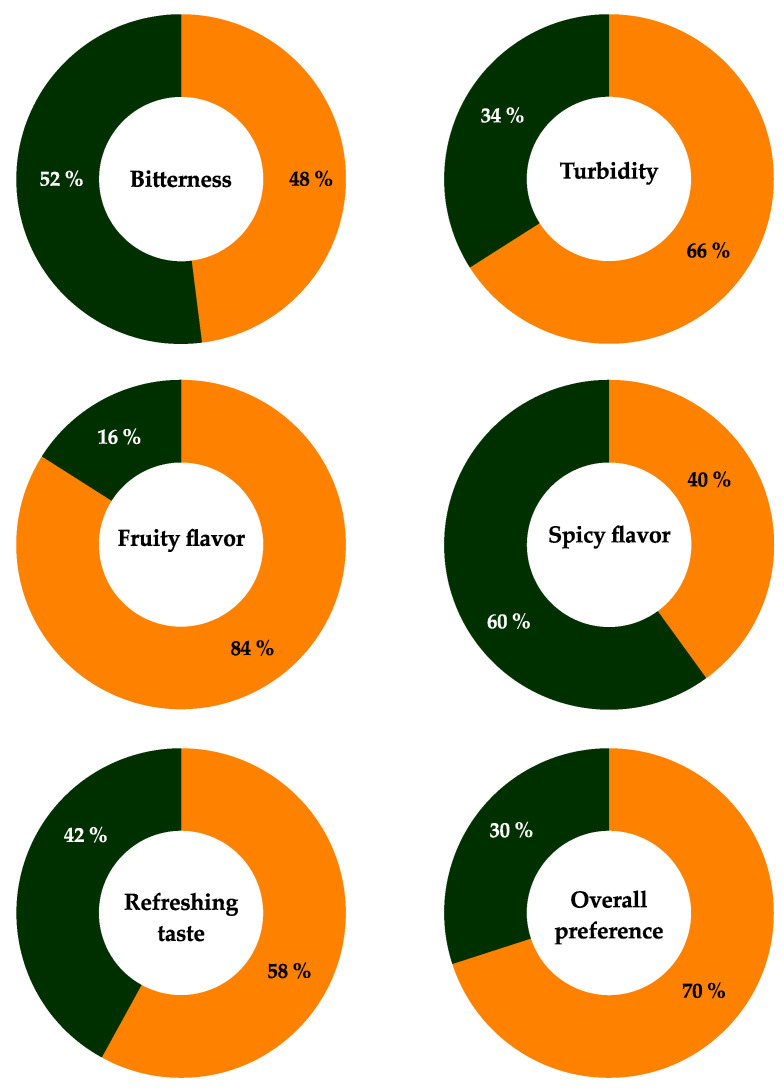
A comparative analysis of various attributes of two Belgian-style pale ales: the product herein elaborated with *P. kudriavzevii* 4A (■) and a commercial reference sample made with *S. cerevisiae* (■).

In the second sensory test, the judges compared various attributes of two Belgian-style pale ales: the present product and a commercial reference sample elaborated with *S. cerevisiae* (Figure 4). The commercial sample had much more perceptible spicy notes and less turbidity than the beer produced in this study. A significant difference existed between the two pale ales in regard to the perception of the panelists of all attributes except bitterness. The beer prepared with *P. kudriavzevii* 4A was found to be fruitier and more refreshing than the commercial sample.

On the whole, it received a greater acceptance by the judges (70%), who perceived notes of an apple and pear flavor, as well as a slight banana aroma. These flavors were probably conferred by the isoamyl alcohol detected in the product. Overall, the current findings indicate that the beer brewed with *P. kudriavzevii* 4A could be attractive to consumers.

## 4. Conclusions

*Pichia kudriavzevii* 4A, a yeast isolated from natural sources, is a novel strain suitable for the elaboration of a Belgian-style pale ale, according to the present results. The yeast was extremely efficient in the consumption of sugars and the generation of ethanol. The barley employed in the brewing process complied with the established standards. Higher alcohols were identified in the resulting beer. The main one, isoamyl alcohol, has been reported to impart fruity flavors to some beverages. A medium level of ethanol was found in the beer (5.2% *v*/*v*), the safety of which was evidenced by the lack of any detectable trace of methanol, mycotoxins, arsenic, or lead. The final product was well accepted by a group of panelists, who judged its sensory characteristics as novel and pleasant, defining it as refreshing and flavorful. The majority preferred the beer elaborated from *Pichia kudriavzevii* 4A to a commercial Belgian pale ale made from *S. cerevisiae*. Hence, it is worthwhile to continue considering *P. kudriavzevii* 4A for the preparation of alcoholic beverages in other reaction systems.

## Figures and Tables

**Table 1:** Physicochemical characteristics of the barley seeds.

Physical Parameters	Results	Acceptable Limit	Reference
Aroma	Good aroma	Good aroma	[25]
Aspect	Good aspect	Good aspect	
Impurities (%)	6.512 ± 0.9	Maximum 10	
Viability (%)	87.33 ± 0.1	Minimum 85	
Weight (kg) per hectoliter	57.17 ± 3.33	Minimum 56	
**Chemical parameters **	**Results **	**Interval of the reported values **	**Reference **
Humidity (%)	8.22 ± 0.04	<12	[34]
Ashes (%)	2.82 ± 0.04 *	2–3.1 *	[45]
Lipids (%)	3.11 ± 0.08 *	1.1–3.1 *	[45]
Crude fiber (%)	6.29 ± 0.01 *	Approx. 6 *	[4]
Crude protein (%)	11.72 ± 0.03 *	8–13.5 *	[4]
Carbohydrates (%)	76.10 ± 0.2 *	72.8–82.8 *	[45]

(*n* = 3; α = 0.05); * evaluated in dry matter.

**Table 2:** Glucose content in the barley seeds at distinct germination times.

Germination Time (days)	Glucose Content (mg g^−1^)
0	1.48 ± 0.05
3	19.50 ± 0.07
4	25.53 ± 0.35
5	25.5 ± 0.20

(*n* = 3; α = 0.05).

**Table 3:** Characteristics of the wort before and after fermentation.

	Before Fermentation	After Fermentation
Total sugars (g L^−1^)	125 ± 1.37	12.9 ± 0.14
Glucose (g L^−1^)	12 ± 0.11	0.0 ± 0.0
Ethanol (g L^−1^)	0.0 ± 0.0	109.18 ± 1.3
Biomass (g L^−1^)	0.1 ± 0.02	44.91 ± 0.60
pH	6.0 ± 0.1	4.5 ± 0.1

(*n* = 3; α = 0.05).

**Table 4:** Physicochemical and microbiological parameters of the beer made with *Pichia kudriavzevii* 4A as the starter culture.

Parameter	Result	Min/Max Limit	Reference
pH (at 20 °C)	4.23 ± 0.1	2.5–5	[53]
Total acidity (mg lactic acid/100 mL anhydrous ethanol)	2.44 ± 0.12	Max. 10	
Ethanol (% *v*/*v*)	5.2 ± 0.2	2–20	
Higher alcohols (mg/100 mL anhydrous ethanol)	140.95 ± 2.2	Not applicable	
Esters (mg/100 mL anhydrous ethanol)	71.72 ± 1.3	Not applicable	
Aldehydes (mg/100 mL anhydrous ethanol)	<LOD ^1^	Not applicable	
Methanol (mg/100 mL anhydrous ethanol)	<LOD ^2^	Max. 300	
Lead (mg L^−1^)	<LOD ^3^	Max. 0.5	
Arsenic (mg l^−1^)	<LOD ^4^	Max. 0.5	
Total coliform bacteria (MPN/100 mL)	<1.1	<1.1	[54]

<LOD, less than the limit of detection; MPN, most probable number; ^1^ 1.07 mg/100 mL anhydrous ethanol; ^2^ 12.6 mg/100 mL anhydrous ethanol; ^3^ 0.30 mg Pb L^−1^; ^4^ 0.002 mg As L^−1^.

**Table 5:** Mycotoxin content of the raw materials and beer.

Mycotoxin	LOD (μg/Kg)	Barley	Malted Barley	Beer	Maximum Allowable Level (μg/kg)	Reference
**Aflatoxins (AFs) **						[64]
Aflatoxin B_1_ (AFB_1_)	1.5	<LOD	<LOD	<LOD	4 (for all AFs)	
Aflatoxin B_2_ (AFB_2_)	1.5	<LOD	<LOD	<LOD		
Aflatoxin G_1_ (AFG_1_)	1.5	<LOD	<LOD	<LOD		
Aflatoxin G_2_ (AFG_2_)	1.5	<LOD	<LOD	<LOD		
**Trichothecenes **						
HT-2	4.71	<LOD	<LOD	<LOD	HT-2 + T-2 ≤ 200	
T-2	2.24	<LOD	<LOD	<LOD		
Neosolaniol (NEO)	1.61	<LOD	<LOD	<LOD	NR	
Diacetoxyscirpenol (DAS)	2.27	<LOD	<LOD	<LOD	NR	
Deoxynivalenol * (DON)	30.0	<LOD	<LOD	<LOD	750	
**Others**						
Ochratoxin A (OTA)	3.0	<LOD	<LOD	<LOD	5 (in cereals); 3 (in cereal products)	
Zearalenone (ZEN)	1.08	<LOD	<LOD	<LOD	1000	
Fumonisin B1 (FUMB1)	100	<LOD	<LOD	<LOD	4000 (FUMB1 + FUMB2)	

LOD = limit of detection; <LOD = below the limit of detection; NR = not regulated; * also known as vomitoxin.

## Data Availability

All relevant data are within the paper.
